# The Effect of Speed of Processing Training on Microsaccade Amplitude

**DOI:** 10.1371/journal.pone.0107808

**Published:** 2014-09-23

**Authors:** Stephen Layfield, Wesley Burge, William Mitchell, Lesley Ross, Christine Denning, Frank Amthor, Kristina Visscher

**Affiliations:** 1 University of Alabama at Birmingham Department of Biology, Birmingham, Alabama, United States of America; 2 University of Alabama at Birmingham Department of Psychology, Birmingham, Alabama, United States of America; 3 University of Alabama at Birmingham Department of Neuroscience, Birmingham, Alabama, United States of America; 4 The Pennsylvania State University, Department of Human Development and Family Studies, State College, Pennsylvania, United States of America; 5 University of Alabama at Birmingham Department of Biomedical Engineering, Birmingham, Alabama, United States of America; 6 University of Alabama at Birmingham Department of Neurobiology, Birmingham, Alabama, United States of America; Barrow Neurological Institute, United States of America

## Abstract

Older adults experience cognitive deficits that can lead to driving errors and a loss of mobility. Fortunately, some of these deficits can be ameliorated with targeted interventions which improve the speed and accuracy of simultaneous attention to a central and a peripheral stimulus called Speed of Processing training. To date, the mechanisms behind this effective training are unknown. We hypothesized that one potential mechanism underlying this training is a change in distribution of eye movements of different amplitudes. Microsaccades are small amplitude eye movements made when fixating on a stimulus, and are thought to counteract the “visual fading” that occurs when static stimuli are presented. Due to retinal anatomy, larger microsaccadic eye movements are needed to move a peripheral stimulus between receptive fields and counteract visual fading. Alternatively, larger microsaccades may decrease performance due to neural suppression. Because larger microsaccades could aid or hinder peripheral vision, we examine the distribution of microsaccades during stimulus presentation. Our results indicate that there is no statistically significant change in the proportion of large amplitude microsaccades during a Useful Field of View-like task after training in a small sample of older adults. Speed of Processing training does not appear to result in changes in microsaccade amplitude, suggesting that the mechanism underlying Speed of Processing training is unlikely to rely on microsaccades.

## Introduction

On average 15 older adults are killed and 500 are injured in vehicular crashes in the US every day [1]. One contributing factor leading to these crashes is a set of cognitive deficits that lead to an increase in driving errors [2] and declines in performance on other tests of everyday activities [3]. These cognitive declines result in a loss of mobility for the older adults [4] which can increase their number of depressive symptoms [5]. Performance on these everyday activities can be predicted by the Useful Field of View (UFOV) test and deficits in everyday activities can be reduced by Speed of Processing (SOP) training [6]. SOP training improves participants' performance on tasks involving simultaneously presented central and peripheral visual stimuli. During training, these stimuli are presented for shorter durations as performance improves. Although SOP training has been shown to increase the processing speed of older adults [7], the mechanisms behind this training are still unclear. Understanding the mechanisms of training is a first step toward developing optimized training paradigms. We hypothesized that microsaccades may be a link between SOP training and the observed benefits.

Microsaccades, small, high velocity eye movements produced 1–2 times per second during fixation [8], are the most important eye movement for restoring visibility to fading targets during fixation [9]. Fading prevention may result from moving the target to a different receptive field and is observed in both central and peripheral vision. Moving a stimulus from one receptive field to another requires larger microsaccadic eye movements in the visual periphery [10] and is associated with better performance on some visual tasks [9], [11]. Changes in oculomotor activity are associated with a participant's task set; in other words, participants change their patterns of eye movements depending on the task they perform [12]. Microsaccades represent an easily modified eye movement that has the potential to strongly influence behavior. We hypothesize that training in a task involving peripheral stimuli may increase the proportion of large microsaccades.

Alternatively, SOP training could result in a decrease in the proportion of large microsaccades. Larger saccadic eye movements have been shown to suppress neural activity [13], [14]. Microsaccades are smaller eye movements which would make smaller changes to visual perception, and, as such, their effect on visual perception is more controversial. However, a recent study has shown microsaccades suppress neural activity during stimulus presentation in primates [15]. We also hypothesize that SOP training will reduce the proportion of large microsaccades to prevent suppression of neural activity, and aid perception of the stimulus. Only larger microsaccades would be suppressed as they have the most impact on peripheral stimuli.

The overall purpose of this experiment is to determine whether the SOP training affects the size of microsaccades. Larger microsaccades would aid performance in peripheral vision due to visual fading or detract from performance due to neural suppression while the stimulus is present. Because microsaccades can also be affected by the anticipation of the stimulus [12], we focus on a particular time frame around the stimulus that includes 450 ms before and after the stimulus is presented. We define this time frame as peristimulus. The overall aim of the work is to determine if SOP training has an effect on the participants' microsaccade magnitude distribution. It was determined that the training had no measureable effect on the distribution.

## Methods

### Ethics Statement

The study was approved by the University of Alabama at Birmingham's Internal Review Board. Written informed consent was obtained prior to study enrollment.

### Participants

**Figure 1:**
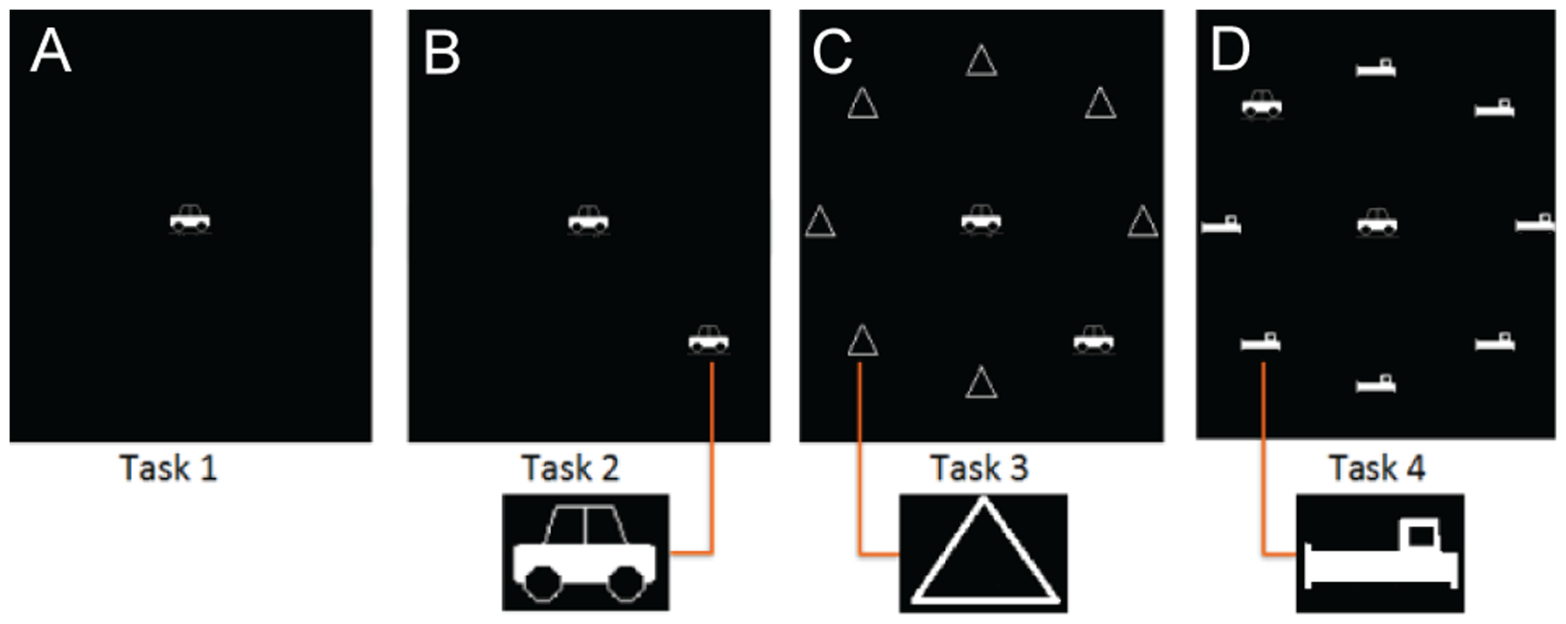
Task. The Useful Field of View-like task consists of 4 task levels. (A) Task 1 presents a central stimulus as car or truck for fixation. (B) Task 2 includes a car (6^°^ eccentricity) peripheral stimulus in one of eight spots near the edge of the screen. Task 3 and 4 are identical to task 2 aside from additional (C) triangle distractors in task 3 and (D) car-like distractors in task 4. Participants respond by clicking on a picture of the central stimulus and a box representing the location of the peripheral stimulus.

Twenty-one volunteers ranging from 65 to 90 years old were included in this study. Eligibility criteria included normal or correctable to normal vision; no evidence or history of dementia; no reports of previous strokes, neurological problems, claustrophobia, steel implants or pacemakers; weight of less than 300 pounds and girth of less than 60 inches; and a 65% or better performance on a modified UFOV task (Figure 1). The participants are a subset of participants from a larger parent study who agreed to participate in eye tracking and who had not previously had cataract surgery.

### Eyetracking Session

A UFOV-like task was created using Psychtoolbox [16]–[18]. It included four stages similar to those of the UFOV: 1. a central stimulus, 2. a central and peripheral stimulus, 3. a central and peripheral stimulus with triangle distractors, and 4. a central and peripheral stimulus with car-like distractors. See Figure 1 for examples of task levels.

The participants were seated and their heads were stabilized with a chinrest approximately 93 cm away from the monitor. The Eyelink 1000 eye tracker was calibrated, and the participants performed the 4 stages of the UFOV-like task [19]. Prior to the stimulus, a fixation cross was presented at the center of the screen. The stimulus in each trial was presented for 450 ms and all task levels contained a central stimulus to ensure continued fixation in the center of the screen. This central stimulus was either a car or a truck, as shown in Figure 1 and subtended approximately 1.25 degrees visual angle. Tasks 2 through 4 also included a simultaneous stimulus in the near periphery, at 6 degrees eccentricity. After the stimulus presentation, white noise was presented for 500 ms and then the participants were asked to identify which of two images was presented as the central stimulus and, when applicable, an additional question with eight options referring to where the peripheral stimulus was located. The participants responded using a mouse which has been shown to be a reliable method [20]. Participants were instructed to keep their eyes fixated on the center of the screen.

The participants performed 25 trials at each task level forming a “block”. The entire test consisted of 20 blocks, 5 at each task level, repeated in the order task 1, task 2, task 3, task 4, task 1, task 2, etc. The eye-tracker was recalibrated at the beginning of each block. The identical test was given at baseline and posttest. Participants performed as instructed during both baseline and post-test, keeping central fixation; no participants made saccades greater than 2.5 degrees during the peristimulus period, meaning they were not looking at the peripheral stimulus located approximately 6 degrees from the center of the screen.

### Training Overview

All participants underwent a screening session and a baseline behavioral session to acquire behavioral measures including vision, mental status, UFOV performance, and neuropsychological measures. Baseline UFOV performance was then used to classify participants as low or high-risk for declines in everyday functioning [21], [22]. After the baseline behavioral session, the participants returned for a pre-training eyetracking session. Participants were randomized to one of three training groups: a SOP training group (n = 7), a social contact control group (n = 6), or a no-contact control group (n = 8). Approximately 5 weeks elapsed between the pre-training and post-training sessions. Following the training period, participants returned for a post-training behavioral session and a post-training eyetracking session.

SOP training involved 5 sessions that were each 2 hours. This computerized training was customized for each participant based on his or her performance. Participants completed tasks that tested their ability to utilize processing speed, divided attention, and selective attention. The training was designed to improve the amount of visual information that an individual could process over brief periods of time. The specific protocol has been described in detail previously [6],[7].

Participants in the social contact control group attended 5 sessions lasting two hours each over approximately 5 weeks during which they performed cognitively stimulating activities (e.g. crossword puzzles, brain teasers).

### Measurement of Microsaccades

Raw gaze position data from the eye tracker output were analyzed using MATLAB analyses described previously [23] to determine microsaccade amplitude and velocity during the 450 ms when the stimuli were displayed (during the stimulus) and 450 ms prior to stimulus presentation (pre-stimulus). Because tasks 2, 3, and 4 involved peripheral stimuli, which are the origin of our hypothesis, we concentrated on these conditions, but for completeness we report the results for task 1 as well. Additionally, previous research has indicated that older adults, regardless of baseline UFOV test performance, tend to do well on task 1, likely a ceiling effect due to that task's simplicity [7]. For completeness, we performed a separate analysis for task 1, and the results are shown in Figure S1.

The microsaccade magnitude distributions were normalized by the number of trials each participant performed, because, for technical reasons fewer than the full 500 trials were recorded for two participants. Normalization meant that, for saccades of a given magnitude, units are in proportion to the number of trials. Paired t-tests were used to detect differences in the distribution with α = 0.05. The microsaccade magnitude distribution of pre and post-tests of each training groups were compared as well as the microsaccade magnitude distribution of pre-tests for high-risk vs. low-risk individuals.

## Results

**Figure 2:**
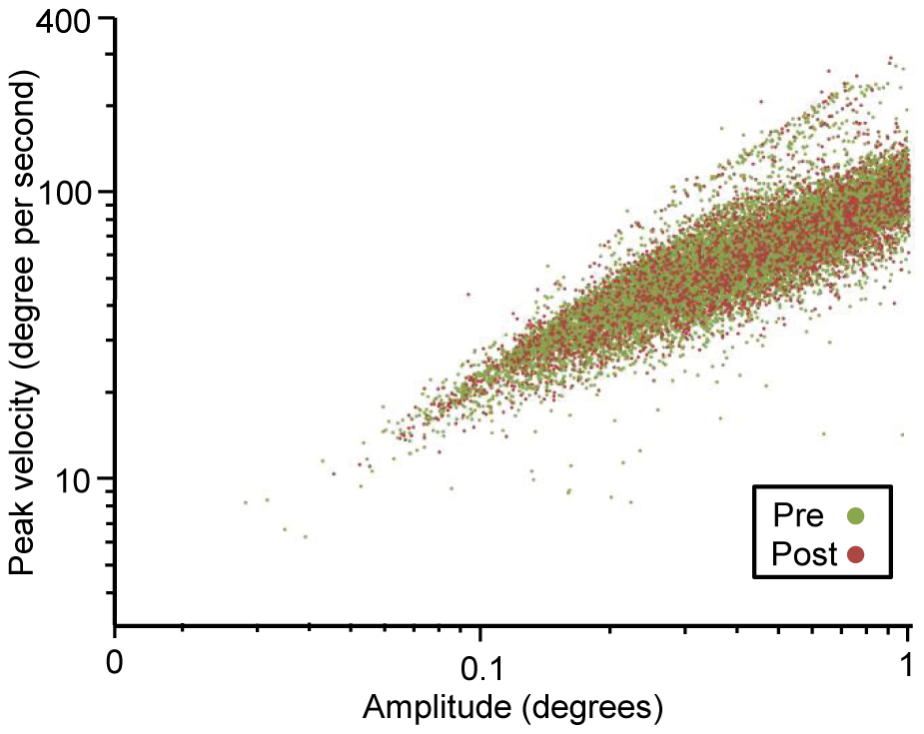
Main Sequence Plot. Comparing amplitude and velocity of eye movements analyzed demonstrates ‘main sequence’ specific to saccadic eye movements.

Microsaccades were defined based on raw eye position data recorded by the eyetracker. Therefore, an analysis was performed to ensure that the eye movements extracted truly represented saccades or microsaccades. Previous literature has shown that the magnitude and peak velocity of microsaccades and saccades are logarithmically related according to a ‘main sequence’ [11]. This relationship (which appears linear on a log-log plot) was observed in our data, confirming that the algorithm did a reasonable job of isolating microsaccades and saccades from our dataset (Figure 2).

**Figure 3:**
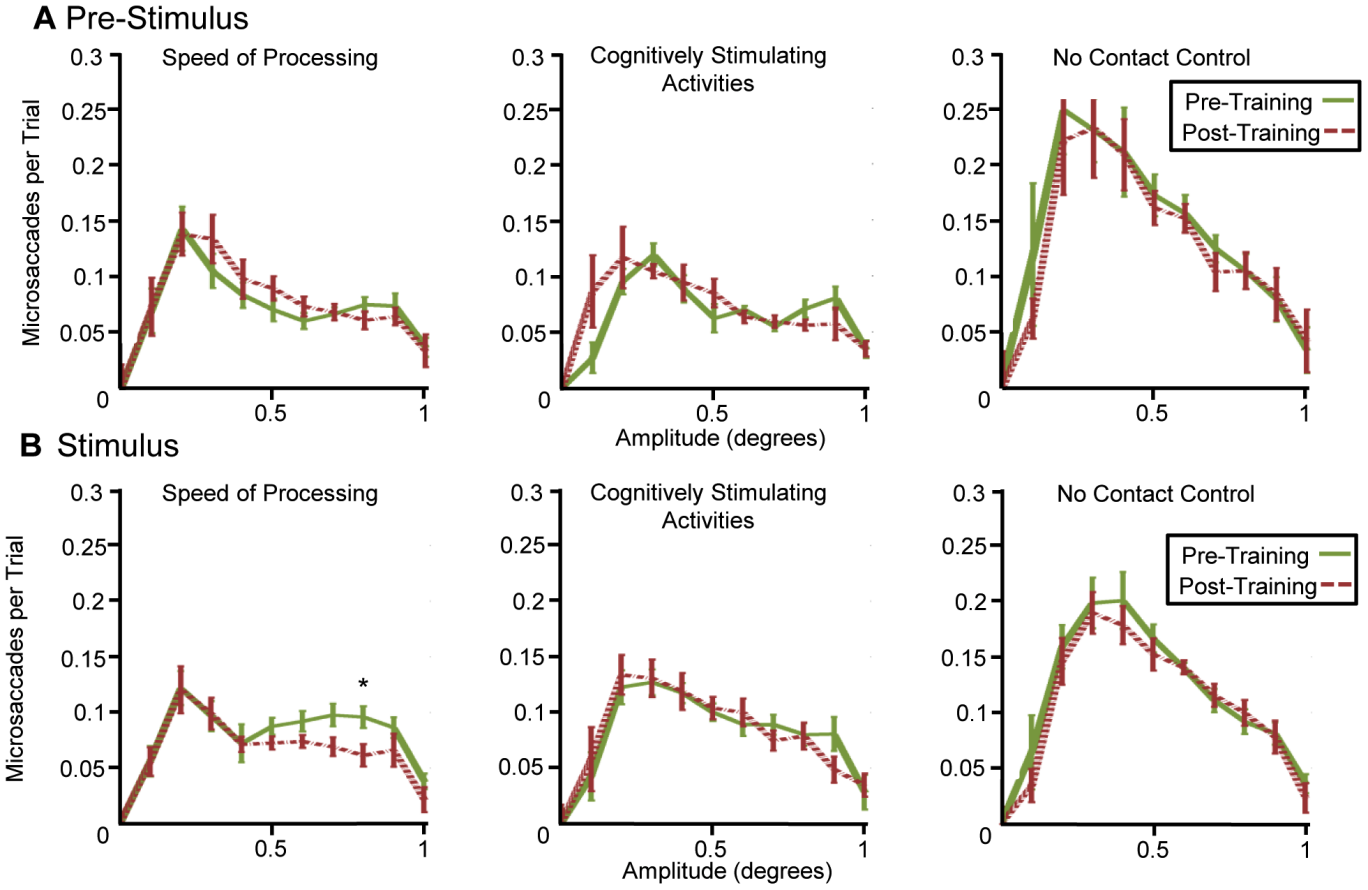
Comparison of Training Groups. Microsaccades were sorted into bins for pre- and post-tests for each group. Within participant standard error of the means are shown and paired t-tests were used to analyze the distribution at each bin. (A) Pre-stimulus data show no significant difference between pre- and post-tests. Data during the stimulus period shows no significant difference in microsaccade amplitude except at 0.8 amplitude in the SOP group (B). This difference was only significant at α = 0.05 prior to multiple comparisons correction but was consistent with the microsaccade suppression hypothesis.

In order to determine whether the distribution of microsaccades changes following training, microsaccades were grouped into bins according to their amplitude. The number of microsaccades per trial, averaged across all participants, is displayed on the x-axis of Figure 3. Error bars displayed on the graph show within-participant standard errors of the mean [24], [25] which are appropriate for assessing differences between pre-and post-tests. T-tests were performed for each bin for each group. This version of testing may result in ‘false positives’ because multiple significance tests are being performed on the same dataset. The method is unlikely to result in ‘false negatives’; that is, the test is relatively unlikely to miss an effect that is present in these data. Comparing pre and post-tests for each training group shows only one difference that meets a threshold of p<0.05 (as discussed, a relatively weak statistical threshold). This difference occurs for microsaccades between the amplitudes of 0.75 and 0.85 in the stimulus time frame (Figure 3B), where pre-training shows slightly more microsaccades per trial than post-training (denoted by *). This direction of result is consistent with the suppression hypothesis, however, the p-value at this point was 0.0498, and does not meet a significant threshold after correction for multiple comparisons (by FDR or Bonferroni correction). For this reason, our data fail to reject the null hypothesis that the distribution of microsaccade amplitudes do not change after training. Our data also fail to reject the microsaccade suppression hypothesis, which predicted that there would be fewer large microsaccades after training. Additionally, as no data showed increases in number or magnitude of microsaccades following training, our data are inconsistent with the hypothesis that training increases the proportion of large microsaccades.

For completeness and in order to avoid effects of a 1 degree maximum cutoff for microsaccades, the same analysis was performed including saccades of all amplitudes. No significant difference was observed between pre and post-tests at larger saccade amplitudes. Also we performed a series of analyses on four demographic variables (age, education, gender, and race) and training group. Table 1 shows that there were no significant demographic differences between the training groups. This shows that the results are unlikely to depend on demographic variables rather than on training group.

**Table 1:** Demographics for training groups.

		Total Sample	SOP Trained	Social Control	No-Contact Control	p-value
Age	Mean	69.19	68.43	68.00	70.75	0.29
	Stdev	3.54	2.51	3.69	4.03	
	Min - Max	65–77	66–72	65–74	66–77	
Education	Mean	25.81	15.57	16.33	15.62	0.78
	Stdev	3.12	3.46	3.14	3.16	
	Min - Max	12–20	12–20	13–20	12–20	
Gender	Male (n)	13	4	5	4	0.42
	Female (n)	8	3	1	4	
Ethnicity	Caucasian (n)	16	6	5	5	0.51
	African American (n)	5	1	1	3	

Statistics for categorical variables (race, gender and education) were assessed with a Pearson's Chi Squared test. Statistics for continuous variables (age) were assessed with ANOVA. P-values are reported.

**Figure 4:**
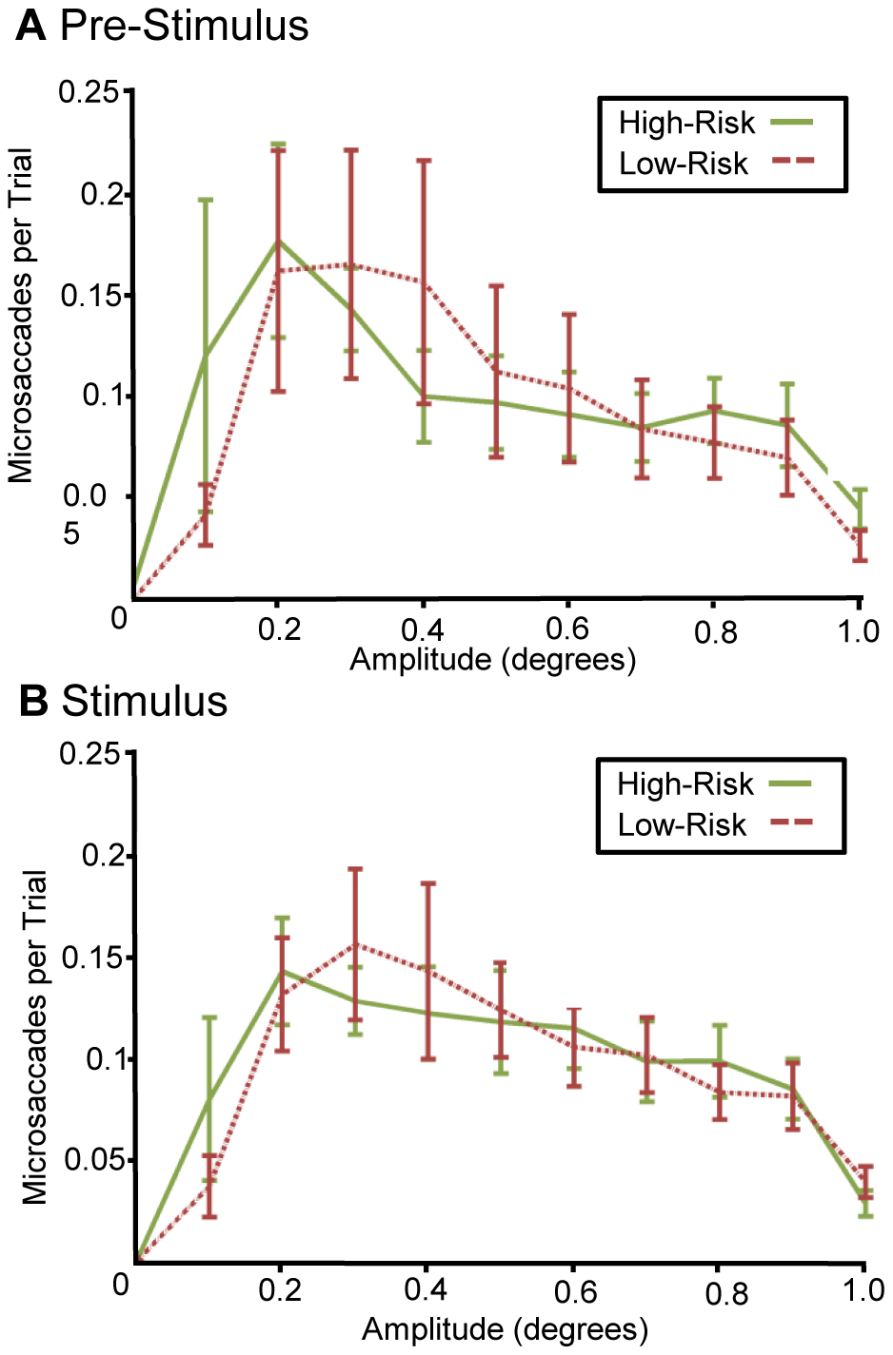
Microsaccades in high and low-risk groups. Participants were designated as high-risk or low-risk based on initial UFOV scores. Pre-test microsaccade magnitude distributions were compared for these groups and no difference was observed in microsaccade magnitude distribution for both pre-stimulus data (A) and data collected during the stimulus (B). Error bars shown are standard error of the mean.

To further detect any relationship between SOP and microsaccade amplitude, we examined whether microsaccade amplitudes differed between participants with good vs. poor SOP performance. Participants were separated into high risk and low risk groups, based on their initial performance on the UFOV [7]. A risk score of 1 to 2 was classified as low-risk and a score of 3 or higher was classified as high-risk. Based on the visual fading hypothesis, we hypothesized that low-risk individuals would produce more, larger microsaccades than the high-risk group because the high-risk group performed poorly. Based on the microsaccade suppression hypothesis, we expected low-risk individuals would produce less, larger microsaccades to reduce neural suppression and increase performance. The two groups displayed no statistically significant difference during the stimulus timeframe, failing to disprove the null hypothesis (Figure 4B). Analyses revealed no significant demographic differences between training groups (Table 1) and no significant demographic differences between the high and low risk groups (Table 2) suggesting that our results are unlikely a function of demographics.

**Table 2:** Demographics for high risk vs. low risk participants.

		High-Risk	Low-Risk	p-value
Age	Mean	69.56	68.92	0.69
	Stdev	3.64	3.60	
	Min - Max	65–77	65–74	
Education	Mean	14.00	17.17	0.13
	Stdev	1.87	3.21	
	Min - Max	12–17	12–20	
Gender	Male (n)	5	8	0.60
	Female (n)	4	4	
Ethnicity	Caucasian (n)	6	10	0.38
	African American (n)	3	2	

Statistics for categorical variables (race, gender and education) were assessed with a Pearson's Chi Squared test. Statistics for continuous variables (age) were assessed with ANOVA. P-values are reported. Additionally, a Pearson's chi-squared test comparing training group by risk category showed an insignificant difference p = 0.823, meaning the training groups included equivalent numbers of each risk category.

As described in the introduction, previous studies have shown that attention influences the rate of microsaccades [10]. In a further set of analyses, we addressed the question of whether training influences microsaccades during preparation for a visual stimulus. The same set of analyses were performed for data obtained in the 450 ms window prior to stimuli presentation (Figure 3A and Figure 4A). There were no significant changes in microsaccade amplitude distribution due to training and no significant differences in microsaccade amplitude between the high risk and low risk participants.

## Discussion

We found that SOP training does not have a statistically significant effect on microsaccade magnitude. The SOP training group was compared to both a no-contact control group and a social contact control group which showed similar results. Prior to multiple comparisons correction, the only statistically significant difference observed was consistent with the microsaccade suppression hypothesis. No data were consistent with the hypothesis that microsaccades counteract visual fading. However, our data fail to reject the null hypothesis that training does not influence microsaccade amplitude. Larger amplitude saccades (above 1 degree) were also not changed with training.

Some previous work has shown that microsaccade rates and amplitudes depend on task difficulty [26]. During a task using auditory stimuli, Siegenthaler and colleagues found that with increasing difficulty, microsaccade rates decreased. The amplitude of those microsaccades increased. We did not find a change in either microsaccade rate or amplitude in our data. However, the trend in the SOP training group toward larger amplitude microsaccades before training is consistent with the suggestion that more difficult tasks are associated with larger amplitude microsaccades.

Caveats and limitations of this study include the fact that this analysis was performed on a relatively small dataset (n = 7, n = 6, and n = 8 respectively), and thus has relatively weak statistical power. Additionally, receptive fields may be too small to affect the distribution of microsaccade amplitudes. It has been shown that the minimum angle of resolution for a 6 degree peripheral stimulus is around 0.04 degrees [27]. This is significantly smaller than microsaccades measured using current techniques. Furthermore, the participants displayed a high percent correct on the UFOV-like task, which suggests the task may have been too easy, reducing the effect of training. Finally, any effects found could be due to speed of saccades rather than size, since they are tightly correlated.

Future research should target other possible mechanisms of SOP training. The short (450 ms) time frame that the stimulus was presented may have been too short for visual fading to occur and too long for saccadic neural suppression to impact perception. Microsaccade suppression may occur during stimulus presentation, but, due to the small sample size, may not be detectable. Additionally the pre-stimulus time period did not show any statistically significant differences between pre and post-test curves. This leads us to believe SOP training does not influence preparation for a stimulus through microsaccade amplitudes. Determining the mechanisms behind SOP training is critical to developing the most efficient and effective interventions to improve the quality of life for many older adults.

## Supporting Information

Figure S1 **Comparison of Training Groups for Task 1.** This figure follows conventions as in Figure 3, but shows data for Task 1 only. Task 1 is separated from the other tasks because this task does not include peripheral stimuli. Microsaccades were collected and sorted into bins for pre- and post-tests for each group. Within participant standard errors of the mean are shown and paired t-tests were used to analyze the distribution at each bin. No significant difference in microsaccade amplitude was found in either pre-stimulus data (A) or data collected during the stimulus (B). (TIF)
