# Supplementary figures and images for: The Effect of Speed of Processing Training on Microsaccade Amplitude
Source: PLoS One. 2014 Sep 23;9(9):e107808. doi: 10.1371/journal.pone.0107808 (PMC4172603; doi:10.1371/journal.pone.0107808)

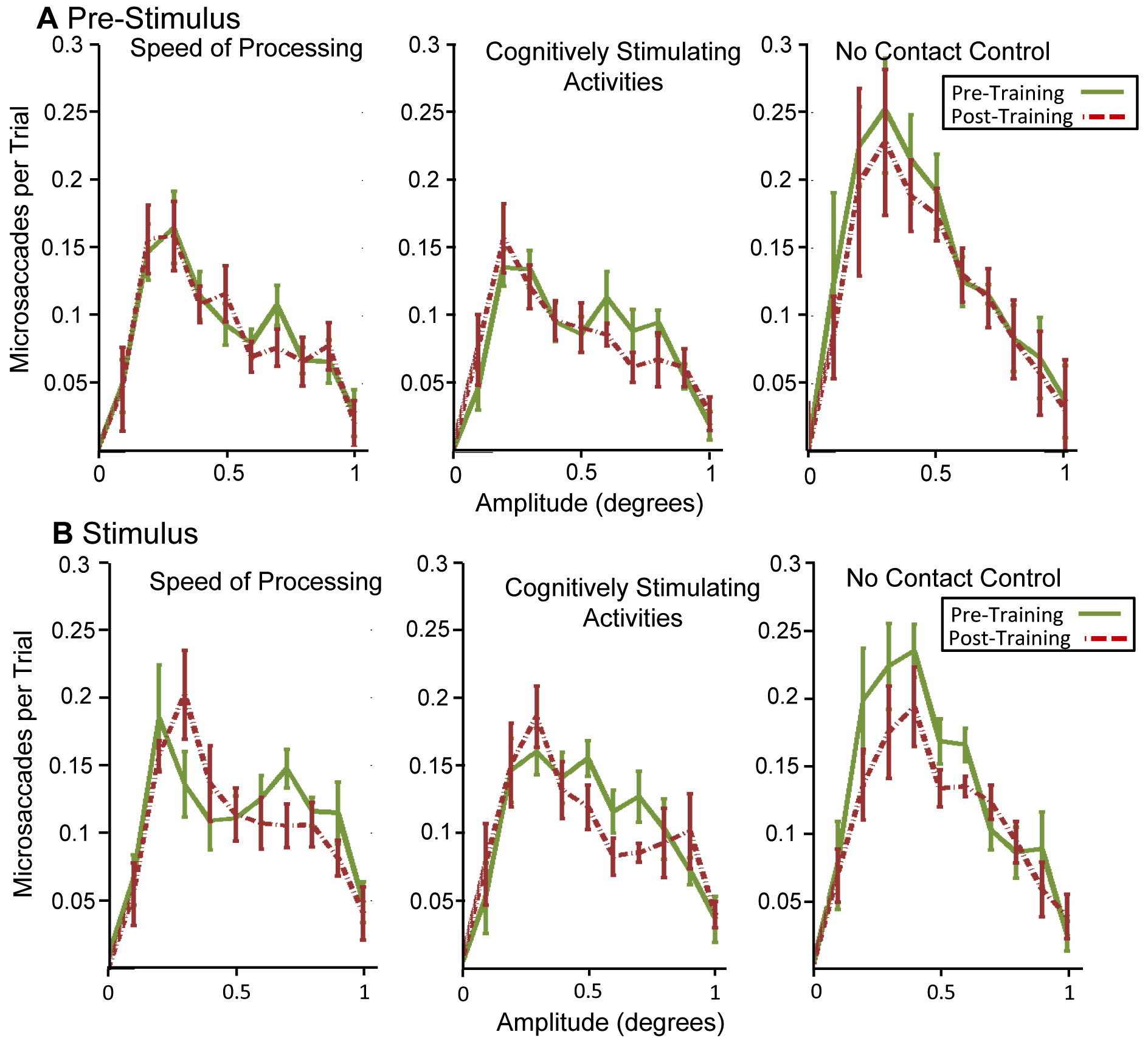

Supplement: Figure S1 — Comparison of Training Groups for Task 1. This figure follows conventions as in Figure 3, but shows data for Task 1 only. Task 1 is separated from the other tasks because this task does not include peripheral stimuli. Microsaccades were collected and sorted into bins for pre- and post-tests for each group. Within participant standard errors of the mean are shown and paired t-tests were used to analyze the distribution at each bin. No significant difference in microsaccade amplitude was found in either pre-stimulus data (A) or data collected during the stimulus (B). (TIF) [file pone.0107808.s001.tif]
